# Prevalence and factors associated with ocular morbidity among prisoners of Luzira prison (Uganda)

**DOI:** 10.1186/s12886-021-02035-w

**Published:** 2021-07-14

**Authors:** Charity Zalwango, Pauline Ayebare, Pius Mwanja, Erima Denis, Moses Kasadhakawo, Micheal Mugerwa, Anne Ampaire

**Affiliations:** 1 Department of Ophthalmology, Makerere University, College of Health Sciences, Kampala, Uganda; 2 Department of Ophalmology, Ruharo Eye Hospital, Mbarara, Uganda; 3 Department of Ophthalmology, Lubaga Hospital, Kampala, Uganda; 4 Department of Ophthalmology, Mulago National Referral Hospital, Kampala, Uganda; 5 Research and Monitoring Department, Aga Khan Hospital, Dar es Salaam, Tanzania

**Keywords:** Ocular morbidity, Inmates, Prison

## Abstract

**Background:**

Globally, ocular morbidity has emerged as a major public concern with at least 2.2 billion people having vision impairment or blindness. Prisoners (inmates) tend to have limited access to health care especially eye health, and as a result some conditions may go undiagnosed or mismanaged. With the increasing prison population in Uganda and in the face of limited facilities, little is known about the prevalence and factors associated with ocular morbidity amongst inmates of Luzira prison.

**Method:**

This was a descriptive cross-sectional study conducted on inmates of Luzira prison. The study included both male (334) and female (33) inmates using the proportionate stratified random sampling. Data on social demographic characteristics, medical, imprisonment factors and ocular assessment was collected using a questionnaire. All complete data was entered using an Epidata version 3.1 entry template, and logistic regression was used to determine associated factors.

**Results:**

Overall, a total of 367 inmates were examined consisting of 334 (91%) males and 33(8.9%) females. The male-to-female ratio was 10:1 with ages ranging from 18 to 76 years. The mean age being 39 years (SD + 13.4) and the overall ocular morbidity was found to be 49%. The most common ocular morbidity included; presbyopia (27.4%), allergic conjunctivitis (19.6%) and cataracts (11.4%). Other disorders included; refractive errors, pterygia, optic atrophy and vitamin A deficiency. There was a statistically significant relationship between ocular morbidity and age (OR 11.96, CI 0.85–2.74), trauma (OR 5.21, CI 1.52–17.87), non-prison food (OR 0.45, CI 0.26–0.79).

**Conclusion:**

The prevalence of ocular morbidity among inmates was found to be high and significantly associated with age, trauma and having meals besides prison food. A fully functional eye unit established within the prison, and timely referral of complicated cases would help in offering quality eye services to the inmates.

**Supplementary Information:**

The online version contains supplementary material available at 10.1186/s12886-021-02035-w.

## Background

Eye diseases have emerged as a major public health concern yet eye care still tends to have a low priority rating in most developing countries’ general health care. WHO statistics show that about 2.2 billion people are visually impaired worldwide, with 1 billion people having moderate-severe visual impairment or blindness.

Ocular morbidity is a widespread term that describes both visually and non-visually impairing eye conditions experienced by a population. It is significant either to the individual (who is concerned enough to seek medical care) or to the professionals (who determine whether the individual would benefit from advice, review or treatment) [1]. Cataracts, refractive errors and glaucoma are major causes of blindness throughout the world and need early detection and timely referral for management [2]. Many non-visually impairing conditions for example; allergic conjunctivitis, presbyopia and dry eye syndrome can cause distress and result in demand for health services [3]. One study showed that presbyopia and refractive errors have significant impact on the performance of both distant and near vision tasks in rural Tanzania [4].

Prisons are places where people are confined for a period determined by law for purposes of retribution, deterrence, rehabilitation and protection of the public or state. A prisoner is a person incarcerated for a crime committed or an individual who is confined against one’s will by force of the law for being a suspect pending investigation and trial for a crime one is accused of. Prisoners have restricted access to health care particularly eye health care, hence eye disorders tend to be high in such communities [5]. In some studies done in Nigerian prisons, a high prevalence of ocular morbidity was shown among the inmates. Olesa prison 69.7% and Benin city 66.5% respectively [5, 6]. Another study was done on the health status of inmates in Italy and eye diseases were some of the most frequently reported problems [7].

Several factors strongly influence the occurrence, burden and pattern of ocular disease in a population. Such factors include; age, socioeconomic, occupational profile and environmental conditions. Health system factors like; accessing health facilities, quality of care and financing also strongly influence the impact of these morbidities [8]. Human Rights Watch study in Ugandan prisons reported that poor conditions (overcrowding, malnutrition, trauma), infectious diseases and inadequate medical care threaten the lives and health of the inmates [9].

The prison population in Uganda is growing at an alarming rate of 10% annually straining the poor prison facilities. It is also estimated that the inmate population will increase from the current 42,000 to the projected 60,000 in the next 5 years if the government does not strengthen crime prevention [10, 11].

Lack of proper awareness about eye health [12], delay in seeking eye care and not so readily available quality eye care services pose a major risk of ocular morbidity. This may result in delayed diagnosis and management of the eye conditions which can result in blindness. In the face of limited resources, it is important to determine the prevalent eye disorders so that there is effective planning for eye care programs for the inmates. The aim of this study was to provide data on prevalent eye diseases and the associated factors among Ugandan inmates. This should be of help in improving eye health care and the prevention of blindness among prisoners.

## Methods

This was a cross-sectional study employing quantitative methods of data collection on inmates of Luzira prison. Approval was granted by the Makerere School of Medicine Research and Ethical Committee and the Commissioner General of Prisons, Uganda Prison Services. Written informed consent was provided by each inmate and all data collected on paper questionnaires was anonymized. This study was carried out according to the principles of the Declaration of Helsinki.

## Quantitative methodology

### Study location and population

This study was carried out at Luzira maximum-security prison in Uganda. Luzira is located in Nakawa division in south eastern Kampala. It is a prison for both males and females. The prison was designed to house 1700 inmates but currently has close to 8000 with over 500 on death row. It has various sections depending on the level of cases namely; Luzira upper prison (maximum security), Murchison bay prison, Remand prison and Luzira women’s prison. By the time of concept and proposal development the prison had 7632 adult male and 700 adult female inmates. Luzira maximum security prison has a referral hospital for all prisons with a dedicated ophthalmic clinical officer, though the ophthalmic clinic lacks the basic diagnostic tools. The inclusion criteria for the study was adult male and female inmates of Luzira prison who consented during the study period.

### Sample size and strategy

Using Kish and Leslie‘s formula an adequate sample size of 361 inmates was obtained. Proportionate stratified random sampling was done using male and female inmates as strata and based on the proportionate contribution to the overall prison population of 91% for males and 8.9% for females, the corresponding sample of 334 males and 33 females was enrolled. The dependent variable was ocular morbidity while the independent variables were: social demographic factors (age, sex, education, previous occupation), imprisonment factors (duration of incarceration, responsibility in prison), associated systemic diseases (HIV, diabetes mellitus), eye/head trauma and social-economic factors (nutrition, drug abuse, alcohol). The non –prison food included other foods besides the posho and beans for example, eggs, meats, vegetables, fruits, etc.

### Data collection

A structured pre-tested questionnaire was administered by a trained research assistant under the direct supervision of the investigator. The respondents who met the inclusion criteria were voluntarily recruited and informed consent obtained before the interview. The questionnaire was used to collect information on; demographic data, duration of incarceration, past and present ocular history, occupation, history of associated systemic diseases, trauma and drug abuse. The inmates’ medical card was used to obtain HIV status while measurements of blood pressure and random blood sugar were taken of all participants on site. A detailed ocular examination was done by the principal investigator and two ophthalmic clinical officers starting with the right eye then the left eye. The visual acuity of the inmates was measured using Snellen’s chart or illiterate ‘E’ chart placed 6 m away from the seated inmate in open daylight by a trained ophthalmic nurse. Each eye was tested separately and unaided. A pinhole was used if V/A was 6/9 and below. Any eye that improved with pinhole by two or more lines was taken to be a case of refractive error and considered for refraction. In prisoners whose refractive errors predated their incarceration, they were tested with their glasses on. Near vision acuity was assessed using a Jaeger chart at 33 cm, and refraction was done on all inmates with impaired vision. The gross visual field assessment was assessed using the confrontation method and compared with the examiner (the examiner had normal visual fields confirmed by perimetry). Extraocular muscle activity was assessed and cover-uncover test was done to assess for phorias. Examination of the lids, conjunctiva, cornea, anterior chamber, pupil and iris was assessed using a pen touch, loupe and a portable slit lamp whereas an amsler grid was used to assess the macular function. Tonometry for intraocular pressure measurements was done using a Perkin’s applanation tonometer after instilling anesthetic drops. Fundoscopy was carried out by direct ophthalmoscope with dilation of pupils whenever a person was found to have a visual acuity less than 6/9. Any anomaly detected during the patient assessment was documented and managed where possible or the patient was referred to Mulago eye clinic for further management.

### Operational definitions

Ocular morbidity was defined as an abnormality in any of the ocular structures which may or may not be visually significant.

Refractive error was diagnosed when the uncorrected visual acuity of 6/9 or worse in any one eye was measured using the snellen chart at a 6-m distance. Pinhole vision was done to differentiate refractive errors from pathological conditions. Presbyopia was defined as binocular presenting near vision <N8 and improving to >N8. Vitamin A deficiency was defined as a history of night blindness or the presence of conjunctival xerosis, bitot spots, corneal xerosis or corneal ulcer on clinical examination. Glaucoma was diagnosed with either two of the features; high intraocular pressure > 21mmhg, significant disc changes (cup: disc > 0.6) or visual field defect. Other ocular diseases such as cataract, conjunctivitis, pterygium, optic atrophy, etc. were diagnosed on clinical examination.

### Data analysis

The collected data was cleaned and exported to Stata 14.0. Continuous variables were summarized using means, standard deviations, medians and ranges. Categorical variables were summarized using frequencies, proportions, percentages and the proportion of prisoners with ocular morbidity was determined. The association between each of the independent factors and ocular disorders was assessed using stepwise logistic regression. The strength of the association was assessed using the odds ratios and 95% confidence intervals of the odds ratio. Variables with a *P*-value of < 0.2 at bivariate analysis were considered for the multivariate model and then entered into a stepwise logistic model. Interactions between the variables which remain in the model were assessed using the chunk test. This was followed by assessing for confounding using a difference of >/ 10% between the crude and adjusted measure of effect (OR) for the variables that would have gone out at each step. Significance was set at a *P*-value of 0.05 or less.

## Results

Three hundred sixty seven inmates participated in the study of which 334(91%) were male. The age of the inmates ranged from 18 to 76 years with a mean age of 39.1(SD + 13.4). More than half of the inmates 193(52.6%) reported having used alcohol at some point in their lives. The majority of the inmates had attained only primary education 181(49.3%) Table 1. All inmates had access to clean water (99.5%). Majority of the inmates (94%) were staying more than 16 persons per cell Table 2. Almost half of the inmates complained of itchy eyes and reduced vision while only 19 of them had a history of trauma. Only eight of them had visual complications from the trauma Table 3. Presbyopia was a significant cause of ocular morbidity (27%). Other morbidities included: allergic conjunctivitis (19.6%) cataracts (10.5%), refractive errors (8.7%) and, vitamin A deficiency (4.6%) as summarised in Table 4.

**Table 1:** Sociodemographic characteristics of the inmates

Variable	n (percentages)
Sex	
Male	334 (91)
Female	33 (8.9)
Occupation	
Business	129 (35.2)
Farming	78 (21.3)
Office related occupation	26 (7)
Others	132 (36)
Alcohol use	
Reported to have taken alcohol	193 (52.6)
Currently take alcohol (*n* = 193)	45 (23.3)
Type of alcohol consumed	
Spirits	86 (44.6)
Beers	124 (64.3)
Local brews	94 (48.7)
Amount consumed (Bottles Per Week)	
1–5	8 (41.5)
5–10	57 (29.3)
> 10	41 (21.2)
Not stated	15 (7.8)
Recreational drugs	118 (32.2)
Cigarette	92 (78)
Raw tobacco	28 (23.7)
Marijuana	41 (34.8)
Cocaine	19 (16.1)
Others	3 (2.5)
Duration of drug use	
Days	3 (2.5)
Months	1 (0.9)
Years	113 (95.8)
Not stated	1 (0.9)
Education level	
Informal	39 (10.6)
Primary	181 (49.3)
Secondary	94 (25.6)
Tertiary	51 (13.9)

**Table 2:** Life of the inmates

Variable	n (percentages)
Ate non prison foods in the last 2 weeks	114 (31.6)
Type of foods (*n* = 114)	
Vitamin A rich foods	86 (75.4)
Energy foods	106 (93)
Body building foods	79 (69.3)
Others	7 (6.1)
Has access to clean water	365 (99.5)
Number of prisoners per cell	
1 to 15	22 (6)
> 16	343 (94.0)
Duration of incarceration	
0–3 months	57 (15.5)
4–12 months	54 (14.7)
1–5 years	114 (31.1)
> 5 years	141 (38.7)
Participant had responsibilities in prison	72 (19.6)
Type of responsibilities (*n* = 72)	
Kitchen staff	7 (9.7)
Warder leader	22 (30.6)
Clinic staff	14 (19.4)
Others	38 (52.8)

**Table 3:** Medical and ocular history of the inmates

Variable	n (percentage)
**Ocular history**	
Wear spectacles	50 (13.6)
Participants reported eye complaints	201 (54.8)
**Eye complaints reported (*n*** **= 201)**	
Itchiness	94 (46.8)
Eye pain	29 (14.4)
Tearing	73 (36.3)
Discharge	7 (3.5)
Reduced distant vision	63 (31.3)
Reduced near vision	91 (45.3)
Foreign body sensation	42 (20.9)
Others	27 (13.4)
History of eye evaluation prior to incarceration	31 (8.5)
History of eye trauma	19 (5.2)
**Duration of trauma (*n*** **= 19)**	
Days	4 (21.1)
Months	2 (10.5)
Years	12 (63.2)
Visual effects from trauma	8 (42.1)
History of surgical operation	3 (0.8)
**Medical history** (*N* = 367)	
Has diabetes	4 (1.1)
Hypertensive	43 (11.7)
Hypertensive on medication	11 (25.6)
Chronic diseases (*n* = 73)	73 (19.9)
HIV	69 (94.5)
Chronic medications (*n* = 73)	68 (91.9)
HIV status	
Positive	72 (19.6)
Negative	293 (79.8)
Unknown status	2 (0.5)

**Table 4:** Pattern of ocular morbidity among inmates

Diagnosis	Frequency	Percentage
Presbyopia	60	27.4
Allergic conjunctivitis	43	19.6
Cataract	23	10.5
Refractive error	19	8.7
Vitamin A deficiency	10	4.6
Pterygium	9	4.1
Optic atrophy	8	3.7
Central corneal scar	3	1.4
Glaucoma	2	0.9
Bacterial conjunctivitis	2	0.9
Pseudophakia	2	0.9
Uveitis	2	0.9
Others	36	17.3

Others include anterior staphyloma, anophthalmos, dry eye, infected lid laceration, lacrimal gland tumour, lid melanoma,old retinal detachment, pinguecula, macular scars, molluscum contangiosum, maculopathy, retinitis

One hundred and eighty inmates (49%) had ocular morbidity in at least one eye and 187 inmates were normal. At multivariate analysis, age of inmate, having food besides prison foods and history of trauma were found to be statistically significant at 95% confidence interval. Inmates who were 50 years and above were twelve times more likely to have ocular morbidity (OR = 11.96, CI 0.85–2.74) than inmates who were of ages18–30. Inmates eating other foods were less likely to have ocular morbidity than those having only prison meals (OR = 0.45, CI 0.26–0.79). Inmates with a history of eye trauma were five times more likely to have ocular morbidity compared to those with no history of trauma (OR = 5.21, CI 1.52–17.87) as summarized in Table 5.

**Table 5:** Multivariable logistic regression for the factors associated with ocular disorders among inmates

	Simple logistic		Multiple logistic	
	OR (95% CI)	*P* Value	OR (95% CI)	*P* Value
**Age**				
18–30 years	1		1	
31–49 years	1.94 (1.17–3.22)	0.011	1.54 (0.87–2.74)	0.140
50 years and above	14.80 (7.28–30.11)	0.001	11.96 (5.31–26.94)	**0.001**
**Occupation**				
Business	1		1	
Farming	1.83 (1.04–3.24)	0.037	0.77 (0.38–1.57)	0.478
Office related occupation	3.02 (1.23–7.47)	0.016	1.44 (0.49–4.27)	0.510
Others	1.16 (0.71–1.88)	0.561	0.99 (0.56–1.75)	0.969
**Food besides prison meals**				
No meal beside prison food	1		1	
Ate food beside prison food	0.63 (0.40–0.99)	0.045	0.45 (0.26–0.79)	**0.006**
**Number of prisoners per cell**				
1 to 4 prisoners	1		1	
5 to10 prisoners	0.27 (0.04–1.79)	0.177	1.05 (0.10–10.74)	0.967
More than 10 prisoners	1.01 (0.25–4.13)	0.980	2.43 (0.45–13.15)	0.302
**Position of responsibility in prison**				
No position of responsibility	1		1	
Has position of responsibility	1.59 (0.95–2.68)	0.080	1.38 (0.73–2.62)	0.319
**Eye trauma**				
No history of eye trauma	1		1	
Has history of eye trauma	4.16 (1.35–12.78)	0.013	5.21 (1.52–17.87)	**0.009**
**Diabetes Mellitus**				
No diabetes	1		1	
Has diabetes mellitus	0.35 (0.04–3.37)	0.361	0.87 (0.06–11.71)	0.915
**Hypertension**				
No hypertension	1		1	
Hypertension	2.82 (1.36–5.88)	0.006	1.12 (0.48–2.62)	0.793

Goodness of fit of the final model: Chi square = 245.51, *p* value 0.515

## Discussion

### Prevalence of ocular morbidity among inmates

In this study, the prevalence was 49% and was higher compared to previously reported studies in Nigeria (26.8%) [13]. The difference could have been brought about by age differences with the Nigerian study reporting a mean age of 27.2 years. The prevalence of eye disease is expected to rise with age [14]. It was also noted that inmates from other prisons would be referred to Luzira prison for various reasons, some being medical or overcrowding in other prisons. This could also probably account for the high ocular morbidity in our study. In comparison to non-incarcerated populations, inmates are known to have elevated rates of morbidities [13].

Similarly to other studies [6, 14, 15], presbyopia, allergic conjunctivitis, refractive errors, cataracts and vitamin A deficiency were among the most common ocular morbidities. Presbyopia was found in 27.4% of the inmates and this prevalence was higher than that of a similar study [15] Ilesa prison Nigeria, a lower prevalence of 10.9% [6] was noted which could be because most of the inmates in Ilesa were in the 21–30-year age group. Presbyopia is a physiological change associated with aging, where the expected age of onset is 40 years with incidence increasing with age and this explains the higher prevalence in this study where more than 50% were aged 40 and above. Identification of presbyopic inmates and providing them with reading glasses will be useful in their rehabilitation and in performance of near tasks.

Allergic conjunctivitis was the second most common morbidity with a prevalence of 19.6% and was comparable to studies done elsewhere [5, 6, 14, 15]. Prisons tend to have similar conditions like overcrowding and being dusty, hence the allergic conjunctivitis which is associated with a lot of discomforts.

Cataracts were found in 23 inmates accounting for 10.9% prevalence which is high in comparison to studies done among Nigerian inmates where prevalence was 5.7% [14] and 4.9% [6]. However, the inmates in Nigeria had a mean age of 27.6 and 32 years respectively and cataract incidence increases with age. In our study, we also found a higher number of trauma cases which could also explain the increased cases of cataracts (traumatic). Cataracts are some of the leading causes of reversible blindness.

The global initiative for the elimination of avoidable blindness (VISION 2020) has recognized refractive errors as a major cause of visual disability. In this study, the prevalence of refractive errors was 8.7% and this was similar to a study done in southwestern Uganda [15].

In comparison to earlier studies done in both southwestern Uganda (35%) and Kenya (23.6%) [15, 16], the prevalence of vitamin A deficiency was low (4.6%). This could be due to the fact that inmates in Luzira prison were routinely given vitamin A supplements and in addition, some inmates had additional meals. Prison food consists mainly of posho (carbohydrate) and beans. Additional meals from relatives can consist of fruits, vegetables, proteins that are rich in vitamin A.

The prevalence of glaucoma among inmates in our study was 0.9% and this is similar to the study done in Mbarara prison that reported 0.4% prevalence [15]. The studies done on the prevalence of glaucoma at Mulago national referral hospital and Ruharo eye hospital were found to be high, 26.1 and 64.2% respectively [17]. These prevalences are much higher than in our study probably due to the fact that Mulago and Ruharo were hospital-based studies with possible referral bias. In this study 2 of the inmates had advanced glaucoma however, they had never had anti-glaucoma treatment. Delayed treatment creates a great danger as there is irreversible visual field loss [18].

Optic atrophy, corneal scars, dry eye syndrome, uveitis and macular scars were documented in these inmates and any of the above conditions may be associated with marked visual impairment especially when there are poor ophthalmic facilities.

### Factors associated with ocular morbidity

In this study, age, trauma and having non prison foods were significantly associated with ocular morbidity.

From this study, the older inmates (> 50 years) were about 12 times more likely to have an ocular morbidity (OR = 11.96, *P*-value =0.001) than those aged 18–30 years. This correlates with findings in the study on the pattern of ocular morbidity in an elderly population which showed a relationship between increasing age and ocular morbidity. This could be due to the physiological changes that happen with aging [1].

Inmates with a history of eye trauma were five times more likely to have ocular morbidity (OR = 5.21, *P*-value = 0.009). A study was done on ocular injuries in patients with major trauma and it was reported that the risk of an eye injury with a facial fracture was six times as that of a patient with none [19]. Inmates are likely to incur trauma when out in the fields, fights with fellow inmates, when being given a punishment or even in the process of arrest or mob justice. Delayed treatment and improper management can lead to visual impairing complications. In a study done in the USA, 16% of the inmates experienced ocular trauma (*p* < 0.001) and 1.2% had open globe injury (*p* = 0.06), requiring surgical intervention [20].

The inmates who had other foods besides prison food were less likely to have ocular morbidity. (OR = 0.45, *P*-value = 0.006c). A diet that includes whole foods containing eye-enhancing nutrients can help to ensure the maintenance of proper vision and eye health. For instance, nutrients like omega 3, vitamin A, C, and zinc contain anti-oxidant properties that lower the risk of some conditions like cataracts, macular degeneration [21]. A study done in Haiti among inmates noted that those who did not receive additional food from visitors were at an increased risk of poor nutritional status and physical health [22].

## Study limitations

- Information bias as some inmates were scared to give information for example in cases of torture or assault.
- Being a cross-sectional study, the ocular disorders found during this season may not be the ones found in other climatic conditions.

## Conclusion

- The prevalence of ocular morbidity among the inmates was high (49%).
- The most common ocular morbidities among the inmates were; presbyopia, allergic conjunctivitis, cataracts, refractive errors and vitamin A deficiency.
- Age, history of trauma and not eating food besides the prison foods were significantly associated with ocular morbidities among inmates.

## Recommendations

- A fully functional eye unit in the prison with essential drugs and equipment. This will help in offering appropriate eye care to the inmates.
- An efficient referral system is advocated for so that those needing secondary or tertiary eye cares can be referred promptly.
- A balanced diet that includes fruits and vegetables as these help with healthy eyes and good vision.

## Supplementary Information

**Additional file 1.** Questionnaire to assess the prevalence and factors associated with ocular morbidity among prisoners of Luzira prison, Uganda, a cross sectional study.

## Data Availability

Data described in the manuscript will be made available by the corresponding author upon request.

## Abbreviations

VA: Visual acuity
HIV: Human immunodeficiency virus
