# Supplementary material for: Prevalence and factors associated with ocular morbidity among prisoners of Luzira prison (Uganda)
Source: BMC Ophthalmol. 2021 Jul 14;21:278. doi: 10.1186/s12886-021-02035-w (PMC8278745; doi:10.1186/s12886-021-02035-w)
Supplement: Supplementary file 1 — Additional file 1. Questionnaire to assess the prevalence and factors associated with ocular morbidity among prisoners of Luzira prison, Uganda, a cross sectional study. [file 12886_2021_2035_MOESM1_ESM.docx]

**DATA COLLECTING TOOL**

Questionnaire to assess the prevalence and factors associated with ocular

Morbidity among inmates of Luzira prison.

Date ……. /………../………. Identity No………….……….

**DEMOGRAPHIC DATA**

A. Age ……………………………………………

B. Sex 1. Male

2. Female

C. Ethnicity: 1. Bantu

2. Nilotic

3. Hamites

4. Nilohamites

5. Others ………………………………………….

D. Previous occupation: …………………………………………………

**SOCIAL AND FAMILY**

a) Have you ever taken alcohol? 1. Yes 2. No

If yes, do you still take alcohol? 1. Yes 2. No

If yes, what type of alcohol?

(i) 1. Spirits 2. Beer 3. Local brews

(ii) How often? Bottles per week: 1[1-5] 2 [5-10] 3 [<10]

b) What is your education level?

1. Informal 2. Primary 3. Secondary 4. Tertiary

2. Have you ever used any drugs? 1. Yes 2. No

If yes.1. cigarette 2.raw tobacco 3. Marijuana 4. Cocaine

Others…………………………………………………………………………

Duration of drug use: 1. Days 2.Months 3.Years

**PRISON LIFE**

1. In the last 2 weeks have you eaten anything besides the prison meals? 1. Yes

2. No

If yes, on how many days did you eat non prison food in the last 2 weeks? .................

What non prison foods did you eat? 1. Vitamin A rich food (fruits, meat, eggs)

2. Energy foods (bread, rice, Irish)

3. Body building foods (beans, peas, fish)

2. Access to clean water 1. Yes 2. No

3. Number of prisoners per cell 1(1-5) 2(6-15) 3(<16)

4. Duration of incarceration 1(0-3) months

2(4-12) months

3 (1-5) years

4 (>5) years

5. Do you have any position of responsibility within prison? 1. Yes 2. No

6. If yes, what’s your responsibility? 1. Kitchen staff 2. Ward leader

3. Clinic staff

4. Others. Specify……………………………………………………

D. **OCULAR HISTORY**

a) Do you wear spectacles or have worn them before? 1. Yes 2. No

b) Do you have any other eye complaint? 1. Yes 2. No 3. Not sure

If yes, what type of complaint?

Itchiness Yes No

Eye pain Yes No

Tearing Yes No

Discharge Yes No

Reduced distant vision Yes No

Reduced near vision Yes No

FB sensation Yes No

Others…………………………………………

c) Did you have an eye evaluation before incarceration? 1. Yes 2. No

d) History of eye trauma 1. Yes 2. No

How long ago? 1. Days 2. Months 3.Years

e) Did you have any visual effect from any trauma? 1. Yes 2. No

f) History of surgical operation on eye? 1. Yes 2. No

**MEDICAL HISTORY**

1. Are you diabetic? 1. Yes 2. No

a) Are you on diabetic medication? 1. Yes 2. No

2. Do you have High blood pressure (Hypertension)? 1. Yes 2. No 3. Not sure

If yes, are you on hypertensive medication? 1. Yes 2. No

3. Do you have a history of any other chronic disease? 1. Yes 2. No

If yes specify…………………………………………

4. Are you on any chronic medication? 1. Yes 2. No

If yes, specify………………………………………………….

5 HIV status 1. Positve 2.Negative 3.Unknown

1. .**EXAMINATION:**

**General examination**

1. Blood pressure (mmHg) ………………….

2. Blood sugar (mmol/l) ……………………

**Ocular examination**

1. Visual acuity RE LE

Pinhole

2. IOP

3. Visual fields

i) Normal

ii) Abnormal

4. Extra ocular muscle activity RE LE

i) Normal

ii) Abnormal

If abnormal specify………………………………………………………………….

5. Whole globe: RE LE

1. Normal
2. Abnormal

If abnormal, specify………………………………………………………………….

6. Eyelids: RE LE

1. Normal

1. Abnormal

If abnormal, specify………………………………………………………………….

7. Conjunctiva RE LE

1. Normal
2. Abnormal

If abnormal, specify……………………………………………………………………

8. Cornea: RE LE

1. Normal

1. Abnormal

If abnormal, specify……………………………………………………………………………

9. Anterior chamber: RE LE

1. Normal

ii) Abnormal

If abnormal, specify…………………………………………………………………

10. Iris RE LE

1. Normal

ii) Abnormal

If abnormal, specify………………………………………………………………………..

11. Pupils RE LE

1. Normal

ii) Abnormal

If abnormal, specify………………………………………………………………

12. Lens RE LE

i) Normal

ii) Abnormal

If abnormal, specify…………………………………………………………………………

11. Vitreous: RE LE

1. Normal

ii) Abnormal

If abnormal, specify……………………………………………………………………………

12. Optic disc: RE LE

1. Normal

1. Abnormal

If abnormal, specify………………………………………………………………………….

13. Retina: RE LE

1. Normal

1. Abnormal

If abnormal, specify……………………………………………………………………………

DIAGNOSIS:……………………………………………………………………………..
